# The Relationships Between Patients’ Demographic Characteristics, Comorbid Diseases, American Society of Anesthesiologists Scores and Inflammation Indexes: A Retrospective Study

**DOI:** 10.4274/TJAR.2025.251959

**Published:** 2025-12-22

**Authors:** Ali Genç, Mehtap Gürler Balta, Vildan Kölükçü, Ahmet Tuğrul Şahin, Yunus Emre Şakacı, Hakan Tapar, Tuğba Karaman, Serkan Karaman

**Affiliations:** 1 Tokat Gaziosmanpaşa University Faculty of Medicine Department of Anaesthesiology and Reanimation, Tokat, Türkiye

**Keywords:** Comorbidity, inflammation, neutrophils, postoperative period, white blood cells

## Abstract

**Objective:**

Parameters that can provide information about patients’ current status are very important in preoperative evaluation. The systemic immune inflammation index (SII), and systemic inflammation response index (SIRI) can be easily calculated with a simple hemogram test, and this testing is frequently requested in preoperative preparation. The aim of this research was to examine the relationship between the SII, and SIRI, along with the demographic characteristics and postoperative clinical course of the patient.

**Methods:**

In the study, the records of patients who presented to the anesthesia outpatient clinic for preoperative preparation were retrospectively reviewed. In this study, the relationships between the SII, and SIRI and each patients’ demographic characteristics, and the American Society of Anesthesiologists (ASA) score, comorbid disease, and length of hospital stay were examined.

**Results:**

For the SII value, there was a statistically significant difference between the ASA1 and ASA2 groups and between the ASA2 and ASA3 groups there was no significant difference between the ASA3 and ASA4 groups (*P* < 0.001, *P* < 0.001, *P*=0.17, respectively). There were statistically significant differences between the ASA1 and ASA2, ASA2 and ASA3, and ASA3 and ASA4 groups for the SIRI value (*P* < 0.001, *P* < 0.001, *P* < 0.001, respectively).

**Conclusion:**

The findings showed relationships between the SII, SIRI, neutrophil-lymphocyte ratio, and platelet-lymphocyte ratio and an increase in patients’ ASA scores. In multivariate analysis, some demographic characteristics of the patients, comorbidities, and the postoperative course were found to be independent risk factors predicting SII and SIRI.

**Main Points**

• Systemic immune inflammation index (SII) and systemic inflammation response index (SIRI), which can be easily calculated from the hemogram test, can reflect the clinical condition of the patient.

• As the American Society of Anesthesiologists score increased, SII and SIRI also increased.

• Some comorbid diseases and postoperative clinical courses of the patients were independent risk factors for increased SII and SIRI.

## Introduction

The neutrophil-lymphocyte ratio (NLR), platelet-lymphocyte ratio (PLR), systemic immune inflammation index (SII = neutrophil X platelet/lymphocyte count) and systemic inflammation response index (SIRI = neutrophil X monocyte/lymphocyte count) can be easily obtained with a simple hemogram test. It has been suggested that these values are a useful parameter regarding the severity of many diseases, but research on this topic is still ongoing.^1^ The immunological response and inflammation have important roles in wound formation and healing. However, increased inflammation may cause undesirable conditions such as tissue and organ damage in the postoperative period.

Preoperative identification of patients scheduled for surgery who have a high risk of complications may provide significant benefits in better management of hospital resources, such as intensive care beds.^2^ The physical status risk classification of the American Society of Anesthesiologists (ASA) is primarily used to identify these patients. Beyond this classification, biomarkers that provide information about the inflammatory processes present in patients may provide additional benefits to the ASA score in risk estimation.^3^ Biomarkers such as B-natriuretic peptide and C-reactive peptide have been used in the past to classify the perioperative risk of patients.^4, 5, 6^ However, it has been reported that these markers do not provide additional benefits in predicting cardiovascular outcomes in patients without heart failure.^4^ Conversely, it has been stated that biomarkers calculated from routine blood tests, such as the NLR and PLR, have better predictive values for death from any cause.^7, 8^ Using biomarkers that can be measured or derived from routinely taken blood samples during a patient’s preoperative preparation phase and have a high predictive value for risk factors is preferable to using biomarkers that require specialized tests.^3^

The NLR is a biomarker that can be used to predict postoperative mortality and morbidity in cardiac and cancer surgeries.^1^ Furthermore, it has been shown to predict morbidity and mortality in patients with acute coronary syndrome.^9^ A high NLR is also associated with an increased risk of mortality after discharge from the hospital following myocardial infarction.^10^ High preoperative values in the elderly have been shown to increase the risk of postoperative cognitive dysfunction.^1^ A high preoperative SII has been shown to be significantly associated with an increased risk of perioperative ischemic stroke.^11^

Systemic inflammation is increasingly accepted as initiating and aggravating the pathological process in chronic diseases.^12^ The relationship between patients’ demographic characteristics and comorbidities and NLR, PLR, SII and SIRI has been investigated in some studies, but conflicting results were obtained. One study showed that the NLR had relationships with hypertension and diabetes mellitus, but no significant relationship was found for asthma, arthritis, age, gender, or obesity.^13^ Furthermore, some studies have shown that obesity, age, chronic lung disease, inflammatory diseases, or smoking are related to these inflammation rates.^12, 14^ It is thought that the use of anti-inflammatory drugs for the treatment of the disease or symptoms, or to prevent pain, may be the cause of these conflicting results.^13^

It is very important to determine the potential risks of patients who are expected to undergo surgery, as well as to implement the necessary perioperative follow-up and treatments.^3^ In addition to the ASA score, the NLR, PLR, SII, and SIRI can provide detailed information about a patient’s underlying inflammatory processes and are easily obtained and calculated; thus, these parameters can be useful for determining perioperative risks and taking the necessary precautions.^3^ In short, these parameters can play a supporting role in preoperative risk estimation and in developing a management plan. The aim of the present study was to investigate the relationship between NLR, PLR, SII and SIRI and chronic diseases, patients’ demographic characteristics, ASA score and length of hospital stay.

## Methods

Before the study began, approval was obtained from the Clinical Research Ethics Committee of Tokat Gaziosmanpaşa University Faculty of Medicine (approval no.: 24-KAEK-207, date: 27.06.2024). In this study, the records of patients aged 18-85 years who presented to the anesthesia clinic for preoperative preparation between 11/01/2023 and 05/01/2024 were examined. Informed consent was obtained from patients. Demographic characteristics, chronic diseases, the ASA score, and the length of hospital stay; neutrophil, lymphocyte, monocyte, basophil, and eosinophil values in the preoperative hemogram sample; and the NLR, PLR, SII, SIRI, hemoglobin, hematocrit, and platelet counts of the patients were recorded. The aim of the study was to examine the relationships between NLR, PLR, SII and SIRI with patients’ demographic characteristics, comorbidities, ASA score, length of hospital stay and outcomes. Patients with any active infection, missing laboratory values, or severe trauma; patients who underwent emergency surgery; and pregnant women were excluded from the study.

### Statistical Analysis

The statistical conformity of the data to normal distribution was evaluated using the One-Sample Kolmogorov-Smirnov test. Qualitative data were presented as numbers and percentages, normally distributed quantitative data were presented as mean and standard deviation, and non-normally distributed quantitative data were presented as median [minimum-maximum (min.-max.)] values. Multiple logistic regression analysis was used to determine the independent predictors of different variables on SII and SIRI. In multiple logistic regression analysis, B (unstandardized coefficient) indicates how much the log-odds of the outcome changes for a one-unit increase in the predictor variable; Beta (standardized coefficient) shows the relative strength of each predictor, allowing comparison across variables measured on different scales; t (test statistic) assess whether the predictor has a statistically significant effect on the outcome. Kruskal-Wallis H test was used to compare SII, SIRI, NLR and PLR values ​​in four ASA groups. When statistically significant differences were found, post-hoc tests with Bonferroni correction were used in pairwise comparisons of groups. The Statistical Package for Social Sciences (version 21.0, SPSS Inc., Chicago, IL, USA) was used to evaluate all data. While analyzing the data, statistical significance was accepted as *P *< 0.05.

## Results

**Figure 1:**
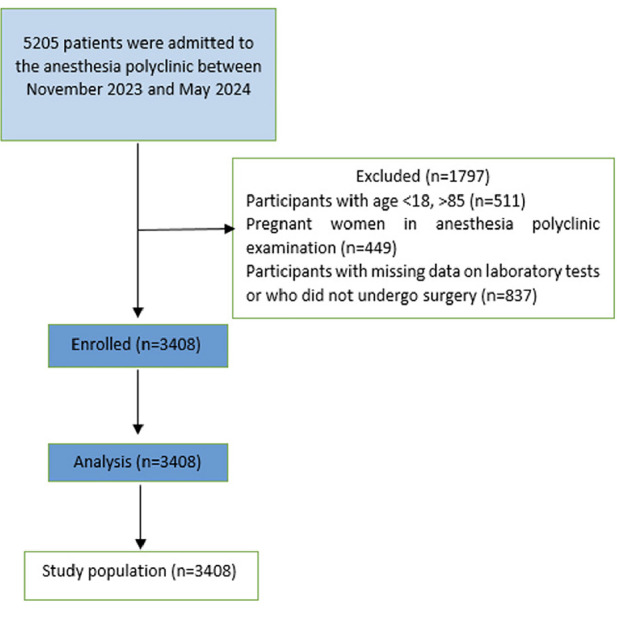
Flow diagram of the study.

A total of 5205 patients were admitted to the anesthesia clinic between 11/01/2023 and 05/01/2024, and after applying the inclusion and exclusion criteria, the records of 3408 patients were reviewed in the current study (Figure 1). The median (min.-max.) age of the patient population was 52 years (18-85), and 53% were female. The demographic data and descriptive characteristics of the patients are detailed in Table 1.

**Figure 2:**
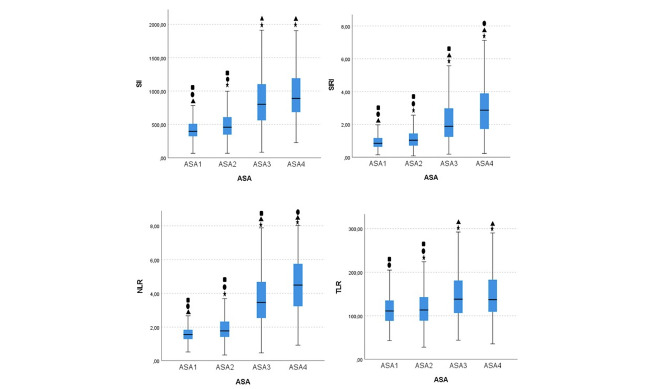
Relationship of the SII, SIRI, NLR and TLR with ASA score. Post-hoc Bonferroni corrected Kruskal-Wallis H test; ✶, statistically significant different from ASA 1; ▲, statistically significant different from ASA 2; ● :, statistically significant different from ASA 3; ■, statistically significant different from ASA 4. ASA, American Society of Anesthesiologists; SII, systemic immune inflammation index; SIRI, systemic inflammation response index; NLR, neutrophil-lymphocyte ratio; PLR, platelet-lymphocyte ratio.

The median (min.-max.) SII values were 394.18 (65.53-1284.72) in ASA1 patients, 456.16 (65.97-2200.83) in ASA2 patients, 801.08 (80.27-4116.8) in ASA3 patients, and 888.76 (224.64-3225.61) in ASA4 patients. There was a statistically significant difference in the SII value between the ASA1 and ASA2, ASA1 and ASA3, ASA1 and ASA4, ASA2 and ASA3, ASA2 and ASA4, but no statistically significant difference was found between the ASA3 and ASA4 groups (*P *< 0.001, *P *< 0.001, *P *< 0.001, *P *< 0.001, *P *< 0.001, *P*=0.17, respectively), (Figure 2).

The SIRI median (min.-max.) values were 0.84 (0.14-4.46) in ASA1 patients, 1.03 (0.08-6.84) in ASA2 patients, 2.51 (0.18-11.99) in ASA3 patients, and 2.86 (0.23-10.5) in ASA4 patients. There were statistically significant differences in SIRI values between the ASA1 and ASA2, ASA1 and ASA3, ASA1 and ASA4, ASA2 and ASA3, ASA2 and ASA4, and ASA3 and ASA4 groups (*P *< 0.001, *P *< 0.001, *P *< 0.001, *P *< 0.001, *P *< 0.001, *P *< 0.001, respectively), (Figure 2).

The NLR median (min.-max.) values were 1.56 (0.53-5.56) in ASA1 patients, 1.78 (0.35-8.01) in ASA2 patients, 3.46 (0.48-9.68) in ASA3 patients, and 4.49 (0.93-10.82) in ASA4 patients. The PLR median (min.-max.) values were 111.21 (18.18-45.08) in ASA1 patients, 113.55 (28.24-420) in ASA2 patients, 138.25 (44-447.62) in ASA3 patients, and 137.23 (35.80-357.32) in ASA4 patients. There was a statistically significant difference in the NLR values of the ASA1 and ASA2, ASA1 and ASA3, ASA1 and ASA4, ASA2 and ASA3, ASA2 and ASA4, and ASA3 and ASA4 groups (*P *< 0.001, *P *< 0.001, *P *< 0.001, *P *< 0.001, *P *< 0.001, *P*=0.02, respectively), (Figure 2). Whereas there was a statistically significant difference between the PLR values of the ASA1 and ASA3, ASA1 and ASA4, ASA2 and ASA3, ASA2 and ASA4, there was no statistically significant difference between the ASA1 and ASA2 or ASA3 and ASA4 groups (*P *< 0.001, *P *< 0.001, *P *< 0.001, *P *< 0.001, *P*=0.1, *P*=1, respectively), (Figure 2).

The relationship between the SII score and demographic characteristics, comorbidities, and the postoperative course of the patients was evaluated using multivariate regression analysis. In the linear regression analysis, the explanatory power of the model was evaluated using the R^2^ value, and it was found that the model explained 37% of the variance in the dependent variable. In addition, the analysis of variance (ANOVA) test result revealed that the model was statistically significant [F(18.3389)=111.95, *P *< 0.001]. In the multivariate logistic regression analysis, gender (male); increasing age, body mass index (BMI) and ASA score; presence of comorbid diseases such as hypertension, diabetes, congestive heart failure, neoplasm, chronic kidney disease; the total length of hospital stay, intensive care unit admission, and the outcome (death) were independent risk factors predicting increasing SII (Table 2). However, there was no relationship between SII and ischemic heart disease, chronic lung disease, thyroid disease, cerebral vascular disease, rheumatic disease, or smoking (Table 2).

The relationships between SIRI scores and demographic characteristics, comorbidities, and the postoperative course of the patients were evaluated using multivariate logistic regression analysis. In linear regression analysis, the explanatory power of the model was evaluated with the R^2^ value and it was found that the model explained 37% of the variance in the dependent variable. In addition, the ANOVA test result revealed that the model was significantly significant [F(18.3389)=92.01, *P *< 0.001]. In the multivariate regression analysis, gender (male); increasing age, BMI, and ASA score; presence of comorbid diseases such as hypertension, congestive heart failure, neoplasm, chronic kidney disease; the total length of hospital stay, and intensive care unit admission were independent risk factors predicting increasing SIRI (Table 3). However, there was no relationship between SIRI and diabetes, ischemic heart disease, chronic lung disease, thyroid disease, cerebrovascular disease, rheumatic disease, smoking, or outcome (Table 3).

The primary outcome of the study was to investigate the relationship between ASA score and systemic inflammation indexes. Patients were divided into four groups based on their ASA scores, and differences in SII levels among these groups were assessed. A post hoc power analysis revealed that the statistical power for the comparison among four groups (n = 3408) exceeded 0.95, with an effect size of f = 0.528, indicating that the sample size was sufficient to detect intergroup differences.

## Discussion

This study showed a significant relationship between the SII, SIRI, NLR, and PLR and the increase in a patients’ ASA score. In multivariate analysis, some demographic characteristics of the patient, comorbidities, and the postoperative course were independent risk factors predicting increased SII and SIRI. Sex, age, BMI, ASA, hypertension, congestive heart failure, neoplasm, chronic kidney disease, total hospital stay, intensive care unit admission, and the outcome (discharge/death) were independent risk factors predicting the SII; conversely, there was no relationship between the SII and ischemic heart disease, chronic lung disease, thyroid disease, cerebral vascular disease, rheumatic disease, diabetes, or smoking. In addition, whereas sex, age, BMI, ASA, hypertension, congestive heart failure, neoplasm, chronic kidney disease, total hospital stay and intensive care unit admission were independent risk factors predicting the SIRI, there was no relationship between the SIRI score and diabetes, ischemic heart disease, chronic lung disease, thyroid disease, cerebral vascular disease, rheumatic disease, smoking, or outcome (discharge/death). In binary logistic regression analysis, ASA scores were independent risk factors predicting congestive heart failure, neoplasm, chronic kidney disease, and chronic lung disease outcome (discharge/death).

Biomarkers that provide information about inflammatory processes in preoperatively determining the group of high-risk patients may provide additional benefits to the ASA score. Tests that can be useful in preoperatively determining high-risk patients scheduled for surgery may provide significant benefits in the effective perioperative management of patients and better management of hospital resources.^2^ Venkatraghavan et al.^3^ showed a strong relationship between the NLR and ASA scores in their study. Moreover, Zhang et al.^11^ stated that there is a relationship between a high SII and increased ASA score. The current study also showed a relationship between ASA scores and increases in the NLR, PLR, SII, and SIRI. In addition, the ASA score was found to be an independent risk factor predicting increased SII and SIRI.

The relationships between inflammation biomarkers and patients’ demographic characteristics and systemic diseases have been investigated in some studies, but conflicting results have been reported. Xia et al.^12^ showed that hypertension, diabetes, obesity, smoking, alcohol use, and physical activity status were associated with high SII and SIRI scores. However, they found that, although age was associated with the SIRI, it was not associated with the SII.^12^ Furthermore, Venkatraghavan et al.^3^ reported that a high NLR was associated with congestive heart failure and malignancy but not with hypertension, diabetes mellitus, chronic kidney disease, ischemic heart disease, or cerebrovascular disease.^3^ Zhang et al.^11^ showed that a high SII was associated with gender, hypertension, diabetes mellitus, history of ischemic stroke, coronary heart disease, renal dysfunction, and peripheral vascular disease but not with age or BMI. In addition, Imtiaz et al.^13^ reported that the NLR had a significant relationship with hypertension and diabetes mellitus but not asthma, arthritis, age, sex, or BMI. However, Furuncuoǧlu et al.^14^ found that BMI significantly affected SII. Lin et al.^15^ reported that SII and SIRI were associated with age but not with gender, hypertension, diabetes, or ischemic stroke. In the present study, sex, age, BMI, ASA, hypertension, congestive heart failure, neoplasm, and chronic kidney disease were independent risk factors predicting increased SII and SIRI, but there was no relationship between the SII and SIRI on the one hand and ischemic heart disease, chronic lung disease, thyroid disease, cerebral vascular disease, rheumatic disease, or smoking on the other. In addition, in this study, diabetes was an independent risk factor predicting increased the SII, but no relationship was found between the SIRI and diabetes.

Previous research has stated that a high NLR predicts mortality in cardiac and vascular surgeries.^16, 17^ It has also been reported that a high NLR is associated with increased morbidity and mortality in the context of sepsis.^18^ In one study, it was stated that high preoperative NLR is associated with increased postoperative morbidity, prolonged intensive care unit stay, and a longer hospital stay.^17^ A high NLR has been shown to be associated with increased tumor necrosis factor alpha and some interleukins [interleukin (IL)-6, IL-7, IL-8, IL-12, IL-17].^19, 20^ Studies have reported that these inflammatory mediators are associated with recurrent ischemic events and poor outcomes in at-risk patients, such as patients with serious heart disease.^21, 22^ In a study on the Kailuan community in Tangshan, China, Jin et al.^23^ revealed that the SII and SIRI are associated with the risk of cardiovascular diseases and death from any cause. Similarly, in a study conducted in a general population, including many ethnicities, Xia et al.^12^ showed that cardiovascular and all-cause mortality risks were associated with high SII and SIRI levels.^12^ Lu et al.^1^ reported that the SII, NLR, and monocyte to lymphocyte ratio were associated with postoperative cognitive decline, a condition that increases the length of the hospital stay and perioperative mortality, as well as cost.^24, 25^ In the present study, total hospital stay and intensive care unit admission were independent risk factors predicting increased SII and SIRI scores. However, while the outcome (discharge/death) was an independent risk factor predicting the SII, there was no such relationship between the SIRI and outcome (discharge/death). In binary logistic regression analysis, independent risk factors predicting the outcome (discharge/death) were the ASA score, congestive heart failure, neoplasm, chronic kidney disease, and chronic lung disease.

### Study Limitations

The limitation of the present study is that it is retrospective, and all patients were from a single center. Inflammation biomarkers were obtained from laboratory tests performed during preoperative anesthesia preparation in the anesthesia outpatient clinic. The study also raises some questions that need to be answered regarding whether changes in systemic inflammation are an effect or a cause of chronic disease, the study could not itself answer them. A prospective study covering a larger general population and dynamically monitoring inflammation biomarkers at different perioperative times may provide more information on this topic.

## Conclusion

In conclusion, the findings of the study showed that there were relationships between the SII, SIRI, NLR, and PLR on the one hand and increases in patients’ ASA scores on the other. In addition, some demographic characteristics of the patient, comorbidities, and the postoperative course were independent risk factors predicting increased SII and SIRI levels. These inflammatory values can be calculated simply and easily from the hemogram test, which is frequently studied in pre-anesthetic evaluation. These parameters, which can reflect a patient’s current condition and provide important information about the clinical course of the patient perioperatively, can play a helpful role in preoperative risk estimation and the development of a management plan. However, we believe that this should be confirmed prospectively by covering a wider general population and by dynamically monitoring inflammation biomarkers at different times perioperatively.

## Ethics

**Ethics Committee Approval:** Ethical approval was obtained from the Clinical Research Ethics Committee of Tokat Gaziosmanpaşa University Faculty of Medicine (approval no.: 24-KAEK-207, date: 27.06.2024).

**Informed Consent:** Informed consent was obtained from patients.

## Figures and Tables

**Table 1. Baseline Characteristics and Clinical Outcomes of the Study Population:** 

Age (years): median (min.-max.)	52 (18-85)
Sex (female/male): n (%)	1791 (53)/1617 (47)
BMI (kg/m^2^): median (min.-max.)	27.47 (15.94-52.85)
ASA score: (I/II/III/IV): n (%)	950/1310/1038/110
Hypertension: n (%)	1285 (38)
Diabetes: n (%)	700 (21)
Ischemic heart disease: n (%)	578 (17)
Congestive heart failure: n (%)	212 (6)
Chronic lung disease: n (%)	580 (17)
Thyroid disease: n (%)	269 (8)
Cerebral vascular disease: n (%)	194 (6)
Neoplasm: n (%)	330 (10)
Rheumatic disease: n (%)	165 (5)
Smoking: n (%)	986 (29)
Chronic kidney disease: n (%)	165 (5)
Total hospital stay (day): median (min.-max.)	3 (0-66)
Intensive care unit admission (yes): n (%)	561 (16)
Outcome (death): n (%)	21 (0.6)
SII: median (min.-max.)	507.44 (63.53-4116.2)
SIRI: median (min.-max.)	1.14 (0.08-11.9)
NLR: median (min.-max.)	2.01 (0.35-10.82)
TLR: median (min.-max.)	119.54 (18.18-450.68)

BMI, body mass index; ASA, American Society of Anesthesiologists; SII, systemic immune inflammation index; SIRI, systemic inflammation response index; NLR, neutrophil-lymphocyte ratio; PLR, platelet-lymphocyte ratio; min.-max., minimum-maximum

**Table 2. Effect of Patient Characteristics and Clinical Outcomes on SII:** 

-	**Unstandardized coefficients**		**Standardized coefficients**	**t**	**Sig.**
-	**B**	**Std. error**	**Beta**	-	-
**Sex (female/male)**	39.018	11.509	0.052	3.390	**<0.001**
**Age (years)**	2.478	0.413	0.117	5.997	**<0.001**
**BMI**	4.707	1.085	0.073	4.338	**<0.001**
**ASA**	63.230	12.307	0.141	5.138	**<0.001**
**Hypertension**	112.876	15.435	0.145	7.313	**<0.001**
**Diabetes**	57.271	14.412	0.061	3.974	**<0.001**
**Ischemic heart disease**	-21.776	19.092	-0.022	-1.141	0.254
**Congestive heart failure**	178.500	25.901	0.114	6.891	**<0.001**
**Chronic lung disease**	23.714	16.202	0.024	1.464	0.143
**Thyroid disease**	3.150	19.857	0.002	0.159	0.874
**Cerebral vascular disease**	38.192	23.690	0.023	1.612	0.107
**Neoplasm**	175.775	19.887	0.138	8.839	**<0.001**
**Rheumatic disease**	-12.193	24.356	-0.007	-0.501	0.617
**Smoking**	12.129	13.096	0.015	0.926	0.354
**Chronic kidney disease**	114.655	26.026	0.065	4.405	**<0.001**
**Total hospital stay (day)**	27.595	2.620	0.223	10.532	**<0.001**
**Intensive care unit admission**	57.352	20.824	0.056	2.754	**0.006**
**Outcome (discharge-death)**	199.163	72.646	0.041	2.742	**0.006**

Multiple logistic regression analysis; Sig. (*P*) < 0.05: statistically significant; BMI, body mass index; ASA, American Society of Anesthesiologists; SII, systemic immune inflammation index

**Table 3. Effect of Patient Characteristics and Clinical Outcomes on SIRI:** 

-	**Unstandardized coefficients**		**Standardized coefficients**	**t**	**Sig.**
-	**B**	**Std. Error**	**Beta**	-	-
**Sex (female/male)**	0.096	0.040	0.038	2.396	0.017
**Age (years)**	0.007	0.001	0.094	4.690	**<0.001**
**BMI**	0.016	0.004	0.075	4.313	**<0.001**
**ASA**	0.281	0.043	0.186	6.568	**<0.001**
**Hypertension**	0.196	0.054	0.075	3.661	**<0.001**
**Diabetes**	0.065	0.050	0.021	1.291	0.197
**Ischemic heart disease**	0.102	0.066	0.030	1.540	0.124
**Congestive heart failure**	0.671	0.090	0.128	7.455	**<0.001**
**Chronic lung disease**	0.056	0.056	0.017	1.003	0.316
**Thyroid disease**	-0.105	0.069	-0.022	-1.528	0.127
**Cerebral vascular disease**	-0.140	0.082	-0.026	-1.703	0.089
**Neoplasm**	0.443	0.069	0.103	6.408	**<0.001**
**Rheumatic disease**	-0.061	0.085	-0.010	-0.719	0.472
**Smoking**	-0.054	0.046	-0.019	-1.191	0.234
**Chronic kidney disease**	0.187	0.090	0.032	2.072	**0.038**
**Total hospital stay (day)**	0.072	0.009	0.173	7.897	**<0.001**
**Intensive care unit admission**	0.318	0.072	0.093	4.394	**<0.001**
**Outcome (discharge-death)**	0.210	0.252	0.013	0.832	0.405

Multiple logistic regression analysis; Sig. (*P*) <0.05: statistically significant; BMI, body mass index; ASA, American Society of Anesthesiologists; SIRI, systemic inflammation response index
